# Systematic literature review of determinants of sedentary behaviour in older adults: a DEDIPAC study

**DOI:** 10.1186/s12966-015-0292-3

**Published:** 2015-10-06

**Authors:** Sebastien F M Chastin, Christoph Buck, Ellen Freiberger, Marie Murphy, Johannes Brug, Greet Cardon, Grainne O’Donoghue, Iris Pigeot, Jean-Michel Oppert, on behalf of the DEDIPAC consortium

**Affiliations:** Institute of Applied Health Research, School of Health and Life Science, Glasgow Caledonian University, Glasgow, UK; Leibniz Institute for Prevention Research and Epidemiology- BIPS, Bremen, Germany; Institute for Biomedicine of Aging Friedrich-Alexander-Universität, Erlangen, Nürnberg Germany; Centre for Physical Activity and Health Research, University of Ulster, Newtownabbey, Co. Antrim, Northern Ireland UK; Department of Epidemiology and Biostatistics and the EMGO Institute for Health & Care Research, VU Medical Centre, Amsterdam, The Netherlands; Department of Movement and Sports Sciences, Ghent University, Ghent, Belgium; Centre for Preventive Medicine, School of Health & Human Performance, Dublin City University, Dublin, Ireland; Institute of Cardiometabolism and Nutrition (ICAN), Centre for Research on Human Nutrition Ile-de-France (CRNH), University Pierre et Marie Curie, Paris, France

**Keywords:** Sitting, Sedentary behaviour, Determinants, Older adults, Ageing, Life-course, Obesity, System-based approach, Physical activity, Environment

## Abstract

**Background:**

Older adults are the most sedentary segment of society and high sedentary time is associated with poor health and wellbeing outcomes in this population. Identifying determinants of sedentary behaviour is a necessary step to develop interventions to reduce sedentary time.

**Methods:**

A systematic literature review was conducted to identify factors associated with sedentary behaviour in older adults. Pubmed, Embase, CINAHL, PsycINFO and Web of Science were searched for articles published between 2000 and May 2014. The search strategy was based on four key elements: (a) sedentary behaviour and its synonyms; (b) determinants and its synonyms (e.g. correlates, factors); (c) types of sedentary behaviour (e.g. TV viewing, sitting, gaming) and (d) types of determinants (e.g. environmental, behavioural). Articles were included in the review if specific information about sedentary behaviour in older adults was reported. Studies on samples identified by disease were excluded. Study quality was rated by means of *QUALSYST.* The full review protocol is available from PROSPERO (PROSPERO 2014: CRD42014009823). The analysis was guided by the socio-ecological model framework.

**Results:**

Twenty-two original studies were identified out of 4472 returned by the systematic search. These included 19 cross-sectional, 2 longitudinal and 1 qualitative studies, all published after 2011. Half of the studies were European. The study quality was generally high with a median of 82 % (IQR 69–96 %) using Qualsyst tool. Personal factors were the most frequently investigated with consistent positive association for age, negative for retirement, obesity and health status. Only four studies considered environmental determinants suggesting possible association with mode of transport, type of housing, cultural opportunities and neighbourhood safety and availability of places to rest. Only two studies investigated mediating factors. Very limited information was available on contexts and sub-domains of sedentary behaviours.

**Conclusion:**

Few studies have investigated determinants of sedentary behaviour in older adults and these have to date mostly focussed on personal factors, and qualitative studies were mostly lacking. More longitudinal studies are needed as well as inclusion of a broader range of personal and contextual potential determinants towards a systems-based approach, and future studies should be more informed by qualitative work.

**Electronic supplementary material:**

The online version of this article (doi:10.1186/s12966-015-0292-3) contains supplementary material, which is available to authorized users.

## Introduction

Too much sitting in particular when accumulated in long uninterrupted bouts is associated with detrimental effects on health and wellbeing, a large number of chronic diseases and all-cause mortality [1–3]. Older adults are the most sedentary segment of society. Sedentary time represents on average 65–80 % of an older adult waking day [4] and over 70 % of older adults spent in excess of 8.5 h per day sitting [5]. This puts older adults specifically at risk of the ill-effects of sedentary behaviour. Indeed, in older adults, sedentary time has been found to be associated with cardiovascular disease [6], frailty, disablement, social isolation [7] and less successful ageing [8]. As the older adult population has increased substantially globally, and it is estimated to reach approximately 22 % of the world’s population by 2050 [9], the public health burden associated with sedentary behaviour is therefore emerging as an important public health concern [7].

Several countries have issued recommendations to reduce sitting time as part of their national physical activity guidelines for older adults [10]. The challenge is to understand and be able to act upon the most effective ways to improve public and individual’s health through interventions and campaigns targeting motivation, ability and opportunity to maintain sedentary time within healthy limits. Identifying determinants of sedentary behaviour and in particular those that are modifiable is a necessary step to develop effective interventions and public health campaigns targeted at reducing sedentary time. This systematic review is one of three reviews which are part of the work performed on sedentary behaviour across the lifecourse in the DEDIPAC study [11]. The aim of this review was to synthesize the current evidence base on the determinants of sedentary behaviour specifically in the older adult’s population.

The current dominant thinking is that determinants of sedentary behaviour can be conceptualised in models such as the ecological model which place the individual within an ecosystem [12, 13] or frameworks that depict the interaction between factors proximal to the individual (biology, psychology, social factors) and distal factors such as environmental, economic, political and socio-economic factors [14]. The secondary aim of this review was to map the current evidence base and knowledge gaps onto these frameworks.

## Review

A common protocol for the three DEDIPAC systematic reviews across the life course (youth, adults, older adults) was developed and is available from PROSPERO (PROSPERO 2014: CRD42014009823).

### Search strategy

The literature search was performed in August 2014 in five electronic databases (Pubmed, Embase, CINAHL with full text, PsycINFO and Web of Science).

The search strategy was based on search terms within four key elements: (a) sedentary behaviour and its synonyms (e.g. sedentariness); (b) determinants and its synonyms (e.g. correlates, factors); (c) types of sedentary behaviour (e.g. TV viewing, gaming) and (d) possible determinants of sedentary behaviour (e.g. environmental, behavioural). Terms referring to these four elements were used as MESH-headings and title or abstract words in all databases. The complete list of search terms is shown in Additional file 1: Table S1. In addition, the reference lists of all included articles were scanned for articles that met the inclusion criteria.

### Selection of studies

To be included in the review, articles had to be published in English l and published between January 2000 and 1^st^ of May 2014. The following study designs were eligible for inclusion; observational studies (cross sectional, case control and prospective), experimental studies (randomized controlled trials, quasi-experimental trials) and qualitative studies. These had to provide information about sedentary time and associated factors for participants aged 65 and over. Articles were included if they measured one or more of the following; total sedentary or sitting time (e.g. minutes per day) or time spent in one or more of the following specific domains of sedentary behaviour; time spent watching TV, screen time, occupational sitting time or motorized transport time. Both objective and subjective measurement outcomes were included. Articles which recruited only specific patient groups or samples identified by diseases were excluded.

The study selection process consisted of three phases: In the initial phase, two reviewers (SC and EF) independently screened articles based on title. In the case of doubt or disagreements, the articles were included in the abstract review phase. In phase two, the abstracts of all articles selected from the initial phase were reviewed and assessed by two independent reviewers (JMO, CB). Any disagreement was resolved by the third reviewer (SC). In the final phase, the remaining articles were fully reviewed by two teams of two reviewers (SC, EF and JMO, CB) using the pre-determined inclusion criteria, and assessed by two independent researchers. Any disagreement between reviewers was solved by discussion in the wider team.

### Data extraction

A standardized template was used to extract data from the included studies using the following heading; General Information - *title of article, main author and publication year*, sample characteristics, study characteristics, measurement of sedentary behaviour and determinants, statistical methods and main results. The four reviewers involved in article selection, extracted data independently (SC,EF and JMO,CB). A quality assurance process enabled cross checking of the data extraction. Discrepancies were resolved through discussion.

### Quality assessment

The quality assessment tool employed was the *QUALSYST* from the *“Standard Quality Assessment Criteria for Evaluating Primary Research Papers from a Variety of Fields” (Alberta Heritage Foundation for Medical Research).* This pragmatic tool incorporates two scoring systems, allowing quality assessment to be conducted on both quantitative and qualitative research [15]. The Qualsyst score is based on eight criteria such as appropriate study design and research question, definition of outcomes and exposures, reporting of bias and confounding, and sufficient reporting of results and limitations. Criteria can be answered as ‘yes’ (2), ‘partial’ (1), ‘no’ (0), and ‘NA’. The Qualsyst score is calculated as sum of ratings of applicable criteria divided by the maximum scores of applicable criteria.

The four reviewers involved in articles selection and data extraction, assessed quality independently (SC, EF and JMO, CB). A quality assurance process enabled cross checking of quality assessment. Discrepancies were resolved through discussion. Articles were not selected based on a threshold of the Qualsyst score.

### Results

**Fig. 1:**
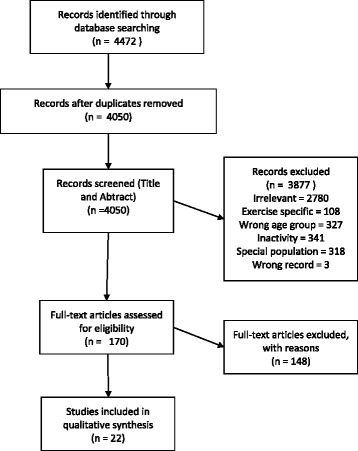
Prisma diagram of the study selection process

Searches of the five databases (Pubmed, Embase, CINAHL with full text, PsycINFO and Web of Science) yielded 4472 records. After duplicates were removed 4050 titles and abstracts were screened against the inclusion criteria. 3877 were excluded for the following reasons; relevance (2780) exercise interventions (108) did not include older adults (327) measured inactivity rather than sedentary behaviour (341) were conducted in special populations (318) or were incorrect records (3). 171 full-text articles were assessed for eligibility with 22 studies meeting the inclusion criteria. Figure 1 illustrates selection of studies from search to inclusion. Table 1 provides an overview of the main characteristics of the studies included.

**Table 1:** Characteristics of studies on determinants of sedentary behaviour in older adults

Author (year)	Country	Design	Participants			Sedentary Behaviour Measure	Potential determinants investigated	Quality Score (%)
			Total	Number (M/F)	Mean (SD) Age or Age Range			
Ku et al., 2011 [24]	Taiwan	Cross-sectional	1450	774 M 676 F	62.1 (9.1)	Sitting time (IPAQ)	Age, Gender, Marital status, Education, Income Wellbeing, Religious belief Employment, living condition Physical activity	91
Lord et al., 2011 [16]	UK	Cross-sectional	56	26 M 30 F	78.9 (4.9)	7 day ActivPAL	Gender, marital status, age, cognitive function, BMI, Depression, Anxiety, Falls, Physical Function, Physical activity, Energy expenditure	86
Balboa-Castillo et al., 2011 [25]	Spain	Longitudinal	1097	40.80 %	70.3 (5.6)	Sitting time (self-report)	Gender, Leisure time Physical Activity	82
Chastin et al., 2012 [31]	UK	Cross-sectional	30	16 M 14 F	M 79.0 (3.6)	Total and pattern of SB by ActivPAL	Gender, Leisure-time Physical Activity, Muscle quality	82
					F 79.3 (3.4)			
Sugiyama et al., 2012 [23]	Australia	Cross-sectional	74788	35721 M 39067 F	5665 M 6552 F ≥ 65 yrs	Time spent sitting in cars in 24 h period	Age	96
Shiroma et al., 2013 [21]	USA	Cross-sectional	7247	Female	71.4 SD (5.8)	Sedentary time by accelerometer	Age, BMI, Smoking	68
Du et al., 2013 [32]	China	Cross-sectional	466,605	188,647 M 277,958 F	51.5 (10.7) yrs	Leisure time SB (self-report)	BMI, Waist circumference, Body Fat	96
Kikuchi et al., 2013 [19]	Japan	Cross-sectional	1655	865 M 800 F	65–74 yrs	TV viewing time (self-report)	Living arrangements, Education, Employment, Self-rated Health, Dog ownership, Driving status, Reported MVPA, Weight	68
Vallance et al., 2013 [23]	Canada	Cross-sectional	375	M	65.3 (7.5)	Sitting time (self-report)	HRQoL, Physical health, Mental health, Global health	91
DeCocker et al., 2013 [51]	Australia	Cross-sectional	4082	1943 M 2139 F	55–65 yrs	TV viewing (self–report)	Education	96
Ishii et al., 2013 [26]	Japan	Cross–sectional	1105	540 M 565 F	40–69 yrs	Leisure screen time (self–report)	Age, Gender, Education, Employment, Marital status, Living arrangement, Income, BMI	82
Arnardottir et al., 2013 [17]	Iceland	Cross–sectional	579	221 M 358 F	73–98 yrs	Sedentary time–accelerometer	Age, Gender, BMI	59
Ikezoe et al., 2013 [40]	Japan	Correlational	19	F	71–96 yrs	Time spent sitting and lying–accelerometers	Muscle strength, Flexibility, Balance, Physical performance	46
Godfrey et al., 2014 [20]	UK	Cross–sectional	98	50 M 48 F	48–89 yrs	Sedentary time by ActivPAL	Age, Gender, Retirement status	96
Larsen et al., 2014 [33]	USA	Cross–sectional	539	135 M 404 F	64.7 (7.48)	Sitting leisure time (self–report)	Ethnicity	77
Barnett et al., 2014 [28]	UK	Longitudinal	3334	1600 M 1734 F	Retired 59.7 (4.7)	TV viewing time (self–report)	SES, Employment status	77
					Non retired 53 (5.1)			
Van der Berg et al., 2014 [27]	Iceland	Longitudinal	565	222 M 343 F	80 (4.7)	Sedentary time–accelerometer	Education, Housing type, Occupation, Smoking, Physical Activity, Weight, BMI, Marital status, Chronic disease and risk	96
Withall et al., 2014 [41]	UK	Cross–sectional	228	117 M 111 F	78.2 (5.8)	Sedentary time–accelerometer	Lower limb function	77
Chastin et al., 2014 [29]	UK	Qualitative	9	F	70–92 yrs	Reasons for sitting and stopping sitting	Pain, Fatigue, Mobility, Ageist stereotypes, Social support/attitudes, Lack of resting places	75
Ortlieb et al., 2014 [52]	Germany	Cross–sectional	191	78 M 113 F	65–89 yrs	Total sedentary time (self–report)	Age	59
Hamrik et al., 2014 [22]	Czech Republic	Cross–sectional	1753	Mixed (48.4 % men)	18–90, 18 % >65	Minutes sitting per day (self–report)	Age, Gender	68
Van Cauwenberg et al., 2013 [18]	Belgium	Cross–sectional	50,986	22,842 M 28,144 F	≥65 years	TV viewing time (self–report)	Age, Gender, Education, Income, Marital status, Walking or Cycling, Functional limitations, Interpersonal relationships. Place attachment, Social participation, Access to and perceived distance of facilities	91

Three of the studies were conducted in North America, 12 in Europe (6 in the UK), five in Asia and two in Australia. The majority of studies employed a cross-sectional design (19) with two prospective cohort studies and one qualitative study.

#### Number and range of participants

Participant numbers ranged from nine in a small qualitative study Chastin et al. [29, 36] to over 460,000 in a cross-sectional study Du et al. [32] and included male and female participants. Most studies used samples of convenience with only one study Ku et al. [24] reporting on a nationally representative sample. Participants were included from a range of SES but tended to be of higher SES and higher educational background. Similarly participants tended to be from urban environments and while they were from a range of ethnicity but no study specifically looked at ethnic minorities. Participants were mostly healthy and community dwelling older adults, however, medical conditions were never mentioned as an exclusion criteria and the samples are therefore likely to include participants with pre-existing morbidities.

#### Quality of studies

Quality scores, expressed as percentage of maximum quality score, ranged from 46–96 % are presented in Table 1.

#### Measurement of sedentary behaviour

Studies included in the review used objective (*n* = 8) (3 activPAL inclinometer, 5 accelerometer) or subjective (self report; *n* = 14) measures of sedentary time. Several studies used proxy self-report measures of sedentary behaviour, including TV or screen viewing (*n* = 4), leisure time sitting, (*n* = 2) sitting in a car (*n* = 1) Sugiyama et al. [23].

Table 2 provides a summary of correlates identified by the selected studies mapped onto Owen’s ecological model. These are discussed in detail below.

**Table 2:** Mapping of the results of the 22 studies onto the ecological model per level and categories and according on whether or not a significant association was found

Level	Category	Correlate	Association with SB	No association with SB
Individual	Demographic	Age	Ku et al., 2011 [24],	Lord et al., 2011 [16],
			Sugiyama et al., 2012 [23],	Arnardottir et al., 2013 [17]
			Shiroma et al., 2013 [21],	
			Ishii et al., 2013 [26],	
			Godfrey et al., 2014 [20],	
			Ortlieb et al., 2014 [34],	
			Hamrik et al., 2014 [22],	
			Van Cauwenberg et al., 2014 [18]	
		Gender	Ku et al., 2011 [24],	Lord et al., 2011 [16],
			Sugiyama et al., 2012 [23],	Balboa–Castillo et al., 2011
			Arnardottir et al., 2013 [17],	[25], Ishii et al., 2013 [26],
			Godfrey et al., 2014 [20],	Hamrik et al., 2014 [22]
			Van Cauwenberg et al., 2014 [18]	
		Marital status	Van der Berg et al., 2014 [27],	Lord et al., 2011 [16],
			Van Cauwenberg et al., 2014 [18]	Ishii et al., 2013 [26]
		Employment / retirement	Kikuchi et al., 2013 [19],	
			Sugiyama et al., 2012 [23],	
			Ishii et al., 2013 [26],	
			Godfrey et al., 2014 [20],	
			Barnett et al., 2014 [28],	
			Van Cauwenberg et al., 2014 [18]	
		Ethnicity	Larsen et al., 2014 [33]	
	Socioeconomic Status	Education	Kikuchi et al., 2013 [19],	Ishii et al., 2013 [26]
			De Cocker et al., 2013 [24],	
			Van der Berg et al., 2014 [27],	
			Van Cauwenberg et al., 2014 [18]	
		Living arrangements	Kikuchi et al., 2013 [19],	Ishii et al., 2013 [26]
			Van der Berg et al., 2014 [27]	
		Income	Sugiyama et al., 2012 [23],	Ishii et al., 2013 [26]
			Van Cauwenberg et al., 2014 [18]	
	Health	Obesity markers	Chastin et al., 2012 [31],	Lord et al., 2011 [16],
			Du et al., 2013 [32],	Ishii et al., 2013 [26],
			Shiroma et al., 2013 [21],	
			Kikuchi et al., 2013 [19],	
			Arnardottir et al., 2013 [17],	
			Larsen et al., 2014 [33],	
			Van der Berg et al., 2014 [27]	
		Quality of Life		Balboa–Castillo et al., 2011 [25], Vallance et al., 2013 [23]
		Heart disease	Van der Berg et al., 2014 [27]	
		Self–rated health		Kikuchi et al., 2013 [19], Vallance et al., 2013 [30]
		Pain	Chastin et al., 2014 [29]	
		Depression		Lord et al., 2011 [16]
		Cognitive characteristics		Lord et al., 2011 [16]
	Psychological	Subjective wellbeing	Ku et al., 2011 [24]	Withall et al., 2014 [41]
		Cognitive Function		Lord et al., 2011 [16]
		Anxiety		Lord et al., 2011 [16]
	Behavioural	Physical Activity	Balboa-Castillo et al., 2011 [25],	Lord et al., 2011 [16]
			Kikuchi et al., 2013 [19],	
			Van Cauwenberg et al. 2014 [18]	
		Smoking	Shiroma et al., 2013 [21]	
		Participation/Volunteering	Van Cauwenberg et al., 2014 [18]	
	Function	Muscle Strength	Ikezoe et al., 2013 [40]	
		Flexibility		Ikezoe et al., 2013 [40]
		Balance	Ikezoe et al., 2013 [40]	
		Functional limitation	Chastin et al., 2014 [29],	
			Van Cauwenberg et al., 2014 [18]	
		Lower limb function	Ikezoe et al., 2013 [40]	Withall et al., 2014 [41]
		Mobility issues		Lord et al., 2011 [16]
Interpersonal	Living with other	Shared accommodation	Kikuchi et al., 2013 [19]	
		Emotional Loneliness	Cauwenberg et al., 2014 [18]	
	People in neighbourhood	Mixed age	Cauwenberg et al., 2014 [18]	
		Perceived number of youth	Cauwenberg et al., 2014 [18]	
		Perceived number of migrants	Cauwenberg et al., 2014 [36]	
Environmental		Driving status	Kikuchi et al., 2013 [19] (female)	Kikuchi et al., 2013 [19] (all)
		Urbanisation	Kikuchi et al., 2013 [19]	
		Living in an apartment	Van der Berg et al., 2014 [27]	
		Access to and perception of facilities	Chastin et al., 2014 [29],	
			Van Cauwenberg et al., 2014 [18]	
		Availability of resting places	Chastin et al., 2014 [29]	

### Individual factors

#### Age

Ten studies examined the association between age and sedentary behaviour. All but two of these studies [16, 17] noted a significant effect of age. van Cauwenberg et al. [18] reported lower TV viewing time by 0.5 min per day for every year after the age of 65. Kikuchi et al. [19] also observed that older adults aged over 70 were less likely to watch TV compared to those aged less than 70 (−0.11 difference in odd ratio). Godfrey et al. et al. [20] reported non-linear association with age, sedentary time was higher by 5 % at 70 years of age but similar at 80 years compared to 65. Shiroma et al., 2013 [21] also observed around 5 % increase in total daily sedentary time per year after the age of 65. Compared to adults Hamrik [22] reported higher sedentary time in older adults (1 h per day). Finally Sugiyama et al., 2012 [23] estimated that older adults spend half the time sitting in cars compared to adults aged between 55–65 years.

#### Sex

Nine studies considered the association between sex and sedentary behaviour with five reporting an association [17, 18, 20, 23, 24] and four failing to note any association [16, 22, 25, 26]. Ku and colleagues [24] and Arnardottir et al. [17] found that males were more sedentary than females. van Cauwenberg et al. [18] found a statistical significant but trivial difference in TV viewing with men reporting 2.9 mins less viewing per day, but Kikuchi et al. [19] reported that men are 21 % more likely to watch TV compared to female. Godfrey et al. [20] found no significant association between gender and total sedentary time but patterns of accumulation of SB differed by gender.

#### Marital status

The association of marital status with sedentary behaviour was equally inconsistent. In a cross-sectional study by Van Der Berg et al. [27], unmarried older adults had higher levels (15.3 mins per day more) of self-reported and objectively measured sedentary behaviour than their married counterparts. This is partially supported by the findings of Van Cauwenberg et al. [18] who reported the highest levels of sedentary behaviour determined by self-reported time spent viewing TV among widows and widowers compared to those who had never married/divorced or were married/cohabiting (6.5 mins per day and 11 mins per day less respectively compared to widowed individuals). However, two studies failed to find a significant association between marital status and sedentary behaviour [16, 26].

#### Employment and retirement status

Employment status showed significant associations with sedentary behaviour in four studies included in this review [19, 20, 27, 28]. Not being in full-time employment (≥35 h per week) almost double the odd ratio of watching TV according to Kikuchi et al. [19]. Mid-life occupation is also associated with sedentary time in old age [27]. Barnett et al. estimated that retirement is associated with higher TV viewing time by 2.6 h per week in white-collars and 3.9 for manual workers [28]. By contrast Godfrey and colleagues [20] reported lower levels of sedentary time in unemployed compared to employed older adults, largely attributable to a greater number of longer bouts of sedentary behaviour among those who were in employment. Similarly van Cauwenberg et al. [18] estimated that having an occupational function through volunteering was associated with almost 15 min less TV time per day. This is consistent with qualitative evidence [29].

#### Educational attainment

Four of the five studies that considered educational attainment found a significant inverse association between level of education and time spent in sedentary behaviours (Table 2) . Estimated effect size reported were 42 min less per day for those with higher education level [18] or 37 % [19] increase in odd ratio for TV time for those with less than university. Ishii et al. [26] did not report a significant association between educational attainments and time spent in sedentary behaviour in Japanese older adults.

#### Health

Eleven of the studies included in this review reported on the relationship between health and sedentary behaviour (Table 2). The majority (*n* = 8) reported inverse associations between a measure of health (psychological, behavioural or functional) and sedentary time. After controlling for socio-demographic factors Ku et al. [24] found an inverse correlation between self-reported sedentary behaviour and subjective well-being with those reporting less sedentary time having higher levels of well-being. This finding was reported by Vallance and colleagues [30] for weekend days but not weekdays with those with the lowest levels of sitting reporting better physical mental and global health than those reporting most sitting at weekends. Seven studies that considered the relationship between sedentary behaviour and obesity found that obese adults reported greater levels of objectively measured [17, 21, 31] and self-reported [27, 32, 33] sedentary behaviour or TV viewing [19]. Estimated effect size reported were 2.5 % [21] and 3.5 % [27] more sedentary time and 50 % higher odd ratio of TV time [19] for obese individuals. van Der Berg et al. [27] is the only study which investigated mid-life health and found that cardiovascular disease have the strongest effect associated with around 7 % higher sedentary time in older age.

The only qualitative study to explore individual reasons for sitting reported physical health problems including pain felt in the standing position, fatigue experienced while standing and functional limitations as the most important reasons for sitting time (Chastin et al. [29, 36].

Two studies showed no association between sedentary behaviour and aspects of self-reported health. Lord et al. [16] found no association between objectively measured sedentary behaviour and self-reported depression, anxiety of cognitive function and Kikuchi et al. [19] found no association between self-reported TV viewing time and self-rated health.

### Interpersonal factors

Only two studies considered interpersonal factors (Table 2). Loneliness was reported as associated with around 2 extra minutes per day of TV time [18] and living alone increase the odd ratio for TV time by 26 % compared to living in shared accommodation [19]. The perception in living in a neighbourhood with not too many older adults but not too many youth or migrant was reported as associated with 5 to 8 min less TV time per day [18].

### Environmental factors

Four of the studies included in this review considered the relationship between environmental factors and sedentary behaviour (Table 2). Kikuchi et al. [19] reported 48 % higher odd ratio for TV time for older adults living in rural area compared to those living in urban settings. On the contrary, the larger study by van Cauwenberg et al. [18] reported 10 min per day higher TV time in urban compared to rural area. In the same study of almost 51,000 Belgian adults it was estimated that the presence of cultural facilities or green spaces in the neighbourhood was associated with less TV viewing by 3 and 3.5 min per day respectively, while perceptions that the environment was unsafe was associated with 3.5 min per day more TV viewing. Living in an apartment or a duplex was associated with 2 % higher levels of objectively measures sedentary behaviour than living in villa our house [18]. When asked about their reasons for sitting, individuals in the study by Chastin et al. [29] reported that a lack of facilities for stimulation and a lack of resting places in the environment encouraged more sitting.

## Discussion

The aim of this review was to summarise current evidence on potential determinants –correlates and predictors- of sedentary behaviour in older adults. With respect to the upcoming challenge to increase public health by reducing sedentary behaviour [7] the knowledge about the determinants of sedentary behaviour will be an important first step for the development of effective strategies. The heterogeneity of these studies in terms of design, samples and measurement methods prevented any form of quantitative synthesis. Therefore this review took a narrative approach informed by a quality assessment with a tool that allows comparison across different type of studies.

To date very few studies have investigated factors which are associated with and may influence sitting time in older adults. The 22 studies included in this review actually provide the sum of total evidence at hand. The current evidence base is therefore extremely limited. This might reflect a lack of interest in this population group [34]. However sedentary behaviour research is a relatively new field, and the oldest study in this review was published only four years ago, which suggest that more studies will be published in the years to come. All the 22 studies were carried out in high income countries. Half of the studies were European, of which half were conducted in the UK. This reflects Europe’s leading role in this domain of research and underscores the value of harmonising European research [11], but also indicates the dearth of information in low and middle income countries.

Within the existing literature, there is extremely limited causal evidence as the vast majority of information comes from quantitative cross-sectional studies, with only two longitudinal studies and one qualitative study available. Consequently, the current evidence base is merely about factors associated or correlated with sedentary time rather than actual determinants per se and little is known about causes of change of sedentary behaviour over time.

Analytically, studies focused mainly on the association between sedentary time and factors through correlation analysis or linear modelling. Only two studies [16, 31] investigated how factors influence the dynamics of sedentary behaviour and how sedentary time is accumulated. Some, for example Barnett et al. [28], investigated the influence of mediators such as socio-economic status, but generally few studies attempted to understand the relationship between factors. This is at odds with current theoretical thinking and frameworks about determinants of healthy lifestyles [13, 14] which point to a complex interplay between proximal and distal factors.

Self-reported measures of sedentary time are easy to administer, inexpensive, do not alter habitual behaviour and are therefore well-suited for large-scale investigations [35]. However, they notoriously underestimate sedentary time but more importantly this error is relative and tends to grow with the amount of time respondents spend sitting [36]. Consequently, association with self-reported measures are distorted [37]. It is therefore reassuring that over one third of the studies used objective means of measuring sedentary time. However there are notable differences amongst objective measures. Only three studies used an inclinometer [20, 31, 38] which directly measure sitting and which has been specifically validated in older adults [39]. Five other studies used accelerometers [17, 21, 27, 40, 41] not specifically validated in this population and which are also known to underestimate sedentary time [42]. One major limitation of objective measures such as inclinometers and accelerometers is that they do not provide contextual information for which context specific questionnaires remain of great help. Importantly, none of the studies combined both objective and self-reported measures as recommended to assess specific domains of sedentary behaviour [35, 43]. Self-reported measures were used to describe sedentary behaviour in four domains defined by the recent taxonomy of sedentary behaviour [43]; leisure time sitting (S8N50), TV viewing (S8Ys5N50), leisure screen time (S8YsN50) and sitting in cars (S712N50). No study deployed objective or subjective means of assessing the context of sedentary behaviour, currently seen as important [44, 45] for understanding the role played by social and physical environmental factors in determining this behaviour.

The evidence currently available might be limited but seems trustworthy as it generally comes from high quality studies. The median quality score was 82 % with an inter-quantile range of 46 to 96 %. A main common feature of the lowest quality studies was that they tended to be secondary data analyses of studies not specifically designed to investigate determinants of sedentary behaviour. Consequently, these often rated low in terms of analysis and in particular regarding the way they controlled for potential confounders. In contrast, studies rated highest were either specifically designed to investigate the determinants of sedentary behaviour or focused their analysis on the association between a single factor and a specific domain of sedentary behaviour. Sample size did not appear to be a determining feature of quality as a number of smaller size studies which were well designed and provided more focused analysis rated higher than some large scale epidemiological studies.

Determinants examined in the articles included in this review were mostly personal factors and information was scarce on other levels of determinants. This means that we currently lack key information about distal determinants of SB in older subjects. Importantly most potential determinants investigated are easy to measure but not modifiable, thus providing little information for intervention studies. In addition, for a number of factors studied the relationship with sedentary behaviour was different depending if sub-domains or total sedentary behaviour time was assessed. This suggests that determinants might be different or have different relationship for different domains of sedentary behaviour. Unfortunately, none of the studies included assessment of several sub-domains. Therefore, potentially important levers for interventions aiming to reduce sedentary behaviour in this specific age group remain difficult to identify at this stage of research.

Among possible determinants assessed, age stands out as the most frequently studied. It was also the potential determinant most frequently associated with sedentary time. In general, age was positively related to increased sedentary behaviour, whether self-reported or measured objectively, and in different countries or regions. Education was also a consistent correlate of sedentary behaviour, with an inverse association in European populations but not in studies from Asia, suggesting a possible cultural factor.

Not being in full employment, being unemployed or retired, was associated with increased sedentary behaviour in reviewed articles. This is also supported by qualitative evidence [29] and results from the largest study in the review [18], which suggests that maintaining an activity or a social role after retirement leads to spend less time sitting. Two papers specifically addressed retirement, one based on screen (or TV) time [28] and the other on objectively sedentary time [20]. The transition to retirement is considered as a major life event in terms of financial as well as behavioural modification, including important changes in sedentary and physical activity behaviours [46, 47]. Retirement leads to a decrease in time constraints possibly corresponding to more free-time available, providing new daily routines and social interactions Barnett et al. [28]. Interestingly, Barnett et al. [28] reported that the largest increase in TV viewing with retirement was observed among manual social classes. This emphasizes the importance of socioeconomic status in midlife for the prediction of sedentary behaviour in old age.

Better health status and lower values for obesity indicators were consistently associated with decreased sedentary time. Interestingly, in the study by Van der Berg et al. [27], a temporal relationship was observed with obesity during midlife and objectively measured sedentary behaviour in old age. This suggests that obesity might be a determinant rather than a consequence of increased sedentary time. In perfect agreement with reports in middle age healthy adults were BMI predicted sedentary time at a five year follow up [48].

It must be noted that health indicators specific to older adults such as functional capacity and markers of geriatric syndromes were rarely assessed [49]. This contrasts with reports from older adults themselves who emphasise the prime importance of physical, and psychological symptoms of geriatric syndromes such as pain, fatigue or fear of falling [29]. Mid-life cardiovascular health appears as a potential determinant of later life sitting behaviour [27].

An important finding of this review is the lack of data on modifiable determinants other than personal factors and the paucity of published qualitative research. Only four out of 22 papers reviewed dealt with potential non-personal or contextual determinants. Although the data suggest possible associations of sedentary behaviour with transportation options [19], the type of housing [27], the presence of cultural facilities in the environment and perceived safety [18] or the availability of places to rest and social isolation [29], a large range of potential determinants at the interpersonal, build environmental and policy level were not addressed. It is therefore difficult to appreciate how the “sedentary space” (by analogy with the concept of activity space [50], might be modified in older subjects. Characteristics of the built environment, as much as social aspects of sitting (e.g. ageist expectation that older adults can only be or even need to be sedentary) should also be further explored before any recommendation can be made. Policy level factors and in particular those that rule the daily working of institutions catering for older adults should be investigated. Older adults themselves report sometimes being forced to sit by staff frightened of them falling Chastin et al. [29, 36].

## Conclusions

In conclusion, results of this review point to the need to explore further potential determinants of sedentary behaviour in older subjects. With respect to the upcoming challenge to increase public health by reducing sedentary behaviour [7], the knowledge about the determinants of sedentary behaviour will be an important first step for the development of effective strategies aiming at decreasing sedentary behaviour in aging populations. Determinants to investigate include not only individual but also contextual such as interpersonal, build or physical environmental and policy determinants according to socio-ecological models of health behaviour [13]. In the future studies need also to focus on modifiable determinants. It should also be stressed that none of the reviewed studies included an analysis of relationships between determinants. Because a majority of studies were cross-sectional, the issue of causality remains elusive. Longitudinal and experimental approaches would be necessary to identify potential levers which could be used to design innovative interventions. The different domains and settings where sedentary behaviour takes place require greater research attention. Improved measures to better capture free-living sedentary activities and their context are needed. More importantly, design of future studies should be informed by qualitative studies and integrate the views and opinions of older subjects themselves in a systems based approach of health promotion through the life course.

## Additional file

Additional file 1: Table S1. Systematic search terms. (DOC 41 kb)

## Abbreviations

SES: Socio-economic status
SB: sedentary behaviour
BMI: Body mass index
